# SIRT1 ameliorates age-related senescence of mesenchymal stem cells via modulating telomere shelterin

**DOI:** 10.3389/fnagi.2014.00103

**Published:** 2014-06-03

**Authors:** Huiqiang Chen, Xianbao Liu, Wei Zhu, Han Chen, Xinyang Hu, Zhi Jiang, Yinchuan Xu, Lihan Wang, Yu Zhou, Panpan Chen, Na Zhang, Dexing Hu, Ling Zhang, Yaping Wang, Qiyuan Xu, Rongrong Wu, Hong Yu, Jian'an Wang

**Affiliations:** ^1^Department of Cardiology, Second Affiliated Hospital, College of Medicine, Zhejiang UniversityHangzhou, China; ^2^Key Lab of Cardiovascular Disease, Second Affiliated Hospital, College of Medicine, Zhejiang UniversityHangzhou, China

**Keywords:** mesenchymal stem cells, SIRT1, aging, senescence, telomerase, shelterin, TPP1

## Abstract

Mesenchymal stem cells (MSCs) senescence is an age-related process that impairs the capacity for tissue repair and compromises the clinical use of autologous MSCs for tissue regeneration. Here, we describe the effects of SIRT1, a NAD^+^-dependent deacetylase, on age-related MSCs senescence. Knockdown of SIRT1 in young MSCs induced cellular senescence and inhibited cell proliferation whereas overexpression of SIRT1 in aged MSCs reversed the senescence phenotype and stimulated cell proliferation. These results suggest that SIRT1 plays a key role in modulating age-induced MSCs senescence. Aging-related proteins, P16 and P21 may be downstream effectors of the SIRT1-mediated anti-aging effects. SIRT1 protected MSCs from age-related DNA damage, induced telomerase reverse transcriptase (TERT) expression and enhanced telomerase activity but did not affect telomere length. SIRT1 positively regulated the expression of tripeptidyl peptidase 1 (TPP1), a component of the shelterin pathway that protects chromosome ends from DNA damage. Together, the results demonstrate that SIRT1 quenches age-related MSCs senescence by mechanisms that include enhanced TPP1 expression, increased telomerase activity and reduced DNA damage.

## Introduction

Mesenchymal stem cells (MSCs) are thought to play important roles in tissue regeneration and repair. This can be handicapped by age-dependent MSCs senescence and loss of function. Previous studies have described an age-dependent decline of MSCs levels in bone marrow and adipose tissue (Stolzing et al., 2008; Alt et al., 2012). Aging negatively affects MSCs proliferation and differentiation including osteogenesis and chondrogenesis (Stolzing et al., 2008; Zhou et al., 2008; Alt et al., 2012). These aging-induced loss of MSCs number and function may be related to the fact that the population of senescent cells shows an age-dependent increase in the stem cell pool (Stolzing et al., 2008; Zhou et al., 2008; Alt et al., 2012). Recent studies indicate that cellular senescence might inhibit cancer early in life but drive age-related phenotypes and associated pathologies later in life (Campisi, 2013; Sikora, 2013). From this perspective, studies focused on age-related MSCs senescence might shed more light on mechanisms of organismal aging and aging-related disease. More importantly, it could potentially provide solutions to reverse or prevent MSCs aging processes.

Sir2p, a NAD^+^-dependent protein deacetylase has been shown to regulate longevity in lower organisms (Frye, 2000), but it is unclear whether SIRT1, the mammalian homolog of Sir2p, has the same effect in humans (Houtkooper et al., 2012). A growing body of evidence suggests that elevated SIRT1 activity can have beneficial effects on aging and aging-related diseases in mammals (Herranz and Serrano, 2010; Herranz et al., 2010; Herskovits and Guarente, 2014). These actions may be related to SIRT1-mediated modulation of DNA and metabolic damage (Herranz et al., 2010; Herranz and Serrano, 2010). Roles for SIRT1 in preventing endothelial cells from replicative senescence or stress-induced premature senescence (SIPS) have been reported recently (Ota et al., 2007; Zu et al., 2010). These anti-aging effects were associated with deacetylation of LKB1 (Zu et al., 2010) or P53 (Ota et al., 2007) by SIRT1. SIRT1 was also shown to protect human umbilical cord fibroblasts from replicative senescence by promoting the transcription of telomerase reverse transcriptase (TERT) (Yamashita et al., 2012). More recently, it was reported that SIRT1 could mitigate the replicative senescence of young MSCs *in vitro* (Yuan et al., 2012). Therefore we postulated here that age-induced biological changes of adult MSCs is driven at least in part by SIRT1.

Therefore, in the present study, we aimed to investigate: first, whether the aging process of MSCs is associated with a down-regulated expression of SIRT1 and whether over-expression of SIRT1 would reverse the phenotype of the age-related MSCs senescence; second, whether cyclin-dependent kinase (CDK) regulators, including P16^INK4A^ (P16) and P21^WAF1^ (P21) are involved in the aging process of MSCs and act as the key mediators of SIRT1 activity through which TPP1 plays the key roles in mediating telomerase function and protects against DNA damage.

## Materials and methods

### Animals

The male Sprague-Dawley (SD) rats were obtained from College of Life Sciences, Zhejiang University (Hangzhou, China). All protocols were approved by the Animal Ethics Committee of Zhejiang University and conformed with the Guide for the Care and Use of Laboratory Animals published by the US National Institute of Health (NIH Publication No. 85-23, revised 1996).

### Isolation and culture of MSCs

Bone marrow cells (BMCs) were harvested according to the protocol of Scutt et al. (Scutt and Bertram, 1995) and MSCs were isolated by methods described previously (Galderisi et al., 2009). Briefly, young (4-week-old) (Stolzing and Scutt, 2006; Khan et al., 2011) and aged (18-month-old) (Khan et al., 2011) male rats were sacrificed and BMCs were obtained by flushing tibias and femurs with culture solution (DMEM supplemented with 10% FBS (Life Technologies, Paisley, UK), 100 U/ml penicillin, and 100 U/ml streptomycin). Cells from one rat were inoculated into two 100-mm dishes with culture solution and non-adherent cells were removed 24 h later. Culture medium was replaced every 3–4 days and the cells were passaged at 70–80% confluence. MSCs at passage 2–5 were used in the following experiments.

### Adipogenic and osteogenic differentiation of MSCs

For adipogenic differentiation, the MSCs were cultured in high-glucose DMEM supplemented with 10% FBS, 1 μ M dexamethasone, 10 μ M insulin, 200 μ M indomethacin and 0.5 mM isobutyl-methylxanthine for 2 weeks (Wu et al., 2010). Differentiated MSCs were evaluated by Oil red O staining. For osteogenic differentiation, the MSCs were cultured in low-glucose DMEM supplemented with 10% FBS, 10 mM β-glycerophosphate, 50 μ g/ml of α-ascorbic acid, and 0.1 μ M dexamethasone for 2 weeks (Li et al., 2011). Differentiated MSCs were identified by alkaline phosphatase staining.

### Flow cytometry analysis

For flow cytometry analysis, young or aged MSCs at passage 3 were washed with phosphate buffered solution (PBS) and labeled for 30 min at 4°C with CD29 (eBioscience, San Diego, CA, USA), CD44H (BD Biosciences, San Jose, CA, USA), CD73 (BD Biosciences, San Jose, CA, USA), CD90.1 (eBioscience, San Diego, CA, USA), CD105 (Abcam, Cambridge, UK), CD31 (Abcam, Cambridge, UK), CD34 (Santa Cruz Biotechnology, Santa Cruz, CA, USA), and CD45 (Santa Cruz Biotechnology, Santa Cruz, CA, USA) antibodies. After washing cells with PBS, cells were analyzed on BD FACSCanto II flow cytometer (BD Biosciences, San Jose, CA, USA).

### Lentiviral vector construction, virus production, and infection

Recombinant lentiviruses containing shRNA against SIRT1 and nonsense shRNA were purchased from Shanghai Sunbio Medical Biotechnology (Shanghai, China). The target sequence for SIRT1 was 5′- GCAGATTAGTAAGCGTCTT -3′ and the negative control sequence was 5′-TTCTCCGAACGTGTCACGT-3′. The lentiviral vector expressing SIRT1 (pLVX-SIRT1-EGFP-3FLAG-Puro) was constructed by cloning the SIRT1 gene into the pLVX-EGFP-3FLAG-Puro vector via EcoRI site, which was also from Shanghai Sunbio Medical Biotechnology (Shanghai, China). The SIRT1 cDNA was amplified using the following primers: Forward: 5′- CTCAAGCTTCGAATTCGCCACCATGGTTGTCGCGTCGCTG-3′, Reverse: 5′-CCATGGTGGCGAATTCATAGTGCTCTGATTTGTCTGGTG-3′.

Viral supernatants was produced in HEK293T cells after cotransfection of pLVX-SIRT1-EGFP-3FLAG-Puro or pLVX-EGFP-3FLAG-Puro vector with the packaging plasmid psPAX2 and the envelope plasmid pMD2.G using Lipofectamine 2000 (Invitrogen, Carlsbad, CA, USA). The supernatant was harvested at 72 h post transfection, filtered through Millex-HV 0.45μ m PVDF filter (Millipore, Billerica, MA, USA), and stored at −80°C until use.

Passages 2–3 of MSCs were infected with the purified lentivirus overnight at the multiplicities of infection (MOI) of 10 with 8 μg/ml polybrene and the medium was replaced with fresh growth medium the day after infection. After puromycin (5 μg/ml) selection, the infection efficiency was determined by EGFP expression. The levels of SIRT1 expression were detected by western blot.

### Senescence-associated β-galactosidase (SA-β-gal) assay

SA-β-gal staining was conducted with senescence β-galactosidase staining kit (Beyotime Institute of Biotechnology, Shanghai, China) per the manufacturer's instructions. Briefly, MSCs were washed with PBS and then fixed with 4% formaldehyde. After washing with PBS, the cells were incubated at 37°C overnight in a humidified chamber with SA-β-gal staining solution. The next day, the cells were washed again in PBS and the percent of senescent cells was examined under the microscope.

### Cellular proliferation test

Cellular proliferation was determined by Cell Counting Kit-8 (CCK-8) (Bestbio, Shanghai, China) according to manufacturer's protocols. In brief, 100 μ l cell suspension (2000 MSCs) with 15% FBS was added into 96-well plate. At 0, 1, 2, 3, 4, and 5 days, 10 μ l CCK8 solution was added to each well. After incubate for 3 h at 37°C, the absorbance was measured at 450 nm by a microplate reader (Bio-Rad, Berkeley, CA, USA).

### Telomere length measurement by real-time PCR

The method measuring telomere length with real-time PCR was reported previously (Peng et al., 2011). Genomic DNA was extracted on the basis of the manufacturer's instrctions (Aidlab, Beijing, China). Quantitative RT-PCR was conducted using SYBR Green Reaction Mix (TaKaRa, Dalian, China) according to the manufacturer's instructions on an ABI PRISM 7900 HT Detection System (Applied Biosystems, Carlsbad, CA, USA). The primers were as follow (Peng et al., 2011): Telomere forward primer 5′-GGTTTTTGAGGGTGAGGGTGAGGGTGAGGGTGAGGGT-3′, reverse primer 5′-TCCCGACTATCCCTATCCCTATCCCTATCCCTATCCCTA-3′. The reference gene 36B4: forward primer 5′-CTCACTCCATCATCAATGGATACAA-3′, reverse primer 5′-CAGCCAGTGGGAAGGTGTAGTCA-3′. PCR conditions were 95°C for 30 s followed by 40 cycles of 95°C for 5 s and 56°C for 31 s (Peng et al., 2011). The single-copy gene 36B4 was used as a control and the target gene expression was calculated using the ΔΔ*Ct* comparative method.

### Quantitative real-time PCR

Total RNA was isolated from MSCs using Trizol reagent (Invitrogen, Carlsbad, CA, USA) per the manufacturer's instructions. The cDNA was synthesized from 1 μ g of RNA using Moloney Murine Leukemia Virus (M-MLV) reverse transcriptase and oligo (dT) 18 primer (TaKaRa, Dalian, China). Quantitative RT-PCR was conducted using SYBR Green Reaction Mix (TaKaRa, Dalian, China) according to the manufacturer's instructions on an ABI PRISM 7900HT Detection System (Applied Biosystems, Carlsbad, CA, USA). PCR conditions were 95°C for 10 min and 40 cycles of 95°C for 30 s, 60°C for 30 s and 72°C for 1 min. PCR primers which were designed using Primer3 Input (version 0.4.0) online software (http://frodo.wi.mit.edu/) are listed in Table 1. Glyceraldehyde-3-phosphate dehydrogenase (GAPDH) was used as a control and the target gene expression was calculated using the ΔΔ*Ct* comparative method.

**Table 1:** **Name and sequence of primer sets for real-time RT-PCR**.

**Gene name**	**Primer sequence**	**Gene ID**	**Product size**
GAPDH F	5′- gagacagccgcatcttcttg-3′	NM_017008.4	224
GAPDH R	5′- tgactgtgccgttgaacttg-3′		
SIRT1 F	5′-cgccttatcctctagttcctgtg-3′	XM_003751934.1	137
SIRT1 R	5′-cggtctgtcagcatcatcttcc-3′		
TERT F	5′-agtggtgaacttccctgtgg-3′	NM_053423.1	232
TERT R	5′-caaccgcaagactgacaaga-3′		
TRF1 F	5′-taccaaactcaagccccatc-3′	NM_001012464.1	170
TRF1 R	5′-gcagcaaactcacatcgaaa-3′		
TRF2 F	5′-agaagaaagcgagtgggtga-3′	NM_001108448.1	178
TRF2 R	5′-ttgtgagtcctgtggctctg-3′		
RAP1 F	5′-gtgagccttggttggaatgt-3′	NM_001013143.1	219
RAP1 R	5′-cctggggaatgggatagttt-3′		
TIN2 F	5′-aaaaccagcatcccacagtc-3′	NM_001006962.1	212
TIN2 R	5′-atggtaggcctgtgttcctg-3′		
POT1 F	5′-cagattcggcagtcactcaa-3′	NM_001024322.1	188
POT1 R	5′-cttcaaaacggcacacaatg-3′		
TPP1 F	5′-cagtgaccacccaggacttt-3′	NM_031357.1	246
TPP1 R	5′-caacaggtcccactccttgt-3′		

### Western blot analysis

Protein extraction and immunoblot analysis were performed according to procedures reported previously with minor modifications (Niagara et al., 2007). In brief, after corresponding treatments, MSCs were rinsed twice with PBS and then were lysed in ice-cold lysis buffer supplemented with protease inhibitors for 30 min. Cell lysates were centrifuged at 14000 g for 30 min at 4°C to remove cell debris. The protein concentrations were determined by the BCA Protein Assay (Bio-Rad, Berkeley, CA, USA). The proteins were separated by 8–12% SDS-PAGE gels and transferred electrophoretically onto polyvinylidene fluoride (PVDF) membranes (Bio-Rad, Berkeley, CA, USA). After non-specific binding site was blocked with 5% non-fat milk, the membranes were rinsed with PBST and incubated with primary antibody (1:1000) overnight at 4°C. After being washed with PBST, the membranes were incubated for 1 h with appropriate HRP- conjugated secondary antibodies (1:5000) at room temperature. After being washed with PBST, protein bands were detected with an ECL kit (Millipore, Billerica, MA, USA) using the Gel Doc EZ Imaging System and analyzed using Image Lab software (Bio-Rad, Berkeley, CA, USA).

Mouse monoclonal anti-SIRT1 antibody, rabbit polyclonal anti-P21 antibody and chicken polyclonal anti-P16 antibody were obtained from Abcam (Abcam, Cambridge, UK). Mouse monoclonal β-actin antibody, horseradish peroxidase (HRP)-conjugated anti-mouse, anti-rabbit and anti-goat secondary antibodies were obtained from Cell Signaling Technology (Beverly, MA, USA). Goat polyclonal TPP1 antibody was purchased from Santa Cruz (Santa Cruz Biotechnology, Santa Cruz, CA, USA). HRP-conjugated anti-chicken second antibody was from Thermo Scientific (Thermo Fisher Scientific, Waltham, MA, USA).

### Immunofluorescence

The cells from various groups were fixed in 4% formaldehyde at room temperature (R.T) for 10 min and permeabilized in 0.5% Triton X-100 in PBS at R.T for 20 min. After washing with PBS, the cells were blocked with 5% bovine serum albumin (BSA) in PBS at R.T for 1 h and thereafter incubated with primary antibody diluted 1:100 at 4°C overnight. Then, after washing with PBS again, the cells were reacted with according secondary antibody (Abcam, Cambridge, UK) at R.T for 1 h. The cells were restained with 4′-6-diamidino-2-phenylindole (DAPI) (Invitrogen, Carlsbad, CA, USA). Fluorescent imaging was conducted with a fluorescence microscope (Leica, Wetzlar, Germany). Mouse monoclonal anti-gamma H2A.X (phospho S139) were obtained from Abcam (Abcam, Cambridge, UK). Rabbit polyclonal anti-TRF2 antibody was purchased from Santa Cruz (Santa Cruz Biotechnology, Santa Cruz, CA, USA).

### Assessment of SIRT1 activity

SIRT1 deacetylase activity was measured in nuclear extracts from MSCs using a histone deacetylase assay kit (AK-555), which was purchased from BIOMOL (Plymouth Meeting, PA, USA) (Solomon et al., 2006). Briefly, enzyme preparations were incubated with 170 μ M NAD^+^ and 100 μ M p53 fluorogenic peptide for 45 min at 37°C followed by incubation in developer for 15 min at 37°C. The fluorescence intensity (excitation at 360 nm and emission at 460 nm) was measured for 1 h every 5 min using a flurorescence plate reader (Molecular Devices, Sunnyvale, CA, USA). SIRT1 deacetylase activity was expressed in relative fluorescence units normalized by protein content.

### Measurement of telomerase activity

Telomerase activity was detected using the Telomeric Repeat Amplication Protocol (TRAP) assay (Roche Applied Science, Indianapolis, IN, USA) per the manufacturer's instructions. In brief, 2 × 10^5^ MSCs were lysed in 200 μ l lysis regent and the cell lysate was centrifuged at 16,000 g for 20 min at 4°C. 0.5 μ g of cell extract was used for PCR reaction in a total volume of 50 μ l. 2.5 μ l of the amplification product was transferred for hybridization and the ELISA assay. Telomerase activity was described as relative telomerase activity (RTA). Each group contained a negative and a high positive control. Telomerase activity was showed as a percentage of the RTA of MSCs relative to the RTA of positive control cells.

### Statistics

All data are expressed as mean ± standard deviation (SD). Analyses were implemented with GraphPad Prism 6.01 software (San Diego, Calif). Comparisons between two groups were tested using Student's *t*-test. A value of *P* < 0.05 was considered significantly different.

## Results

### Characterization of young and aged MSCs

**Figure 1:**
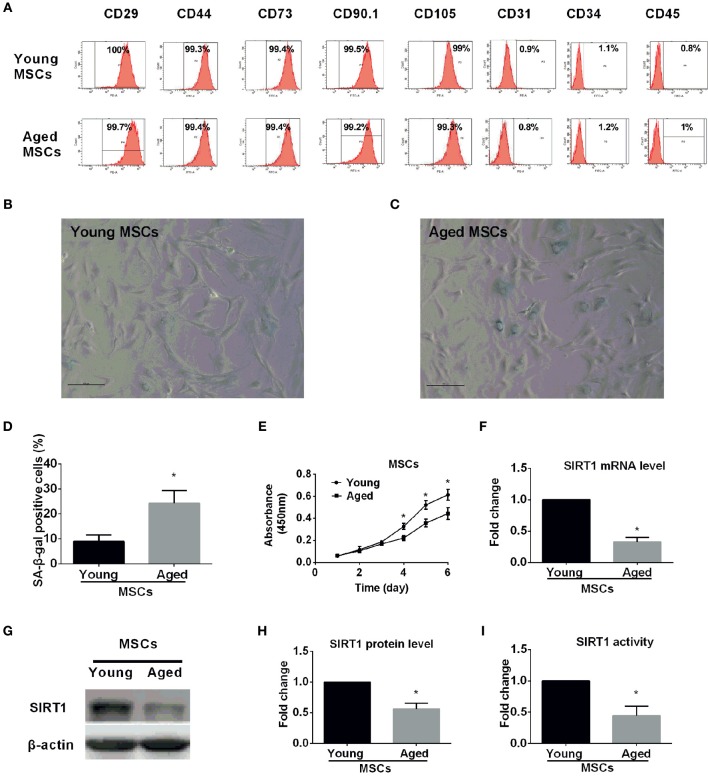
**Characterization of young and aged MSCs. (A)** Phenotypic characterization of young and aged MSCs by flow cytometry. Representative results showed that both young and aged MSCs were both positive for CD29, CD44, CD73, CD90, and CD105 and negative for CD31, CD34, and CD45. β-galactosidase (SA-β-gal) staining was performed for young **(B)** and aged **(C)** MSCs, with percentage of SA-β-gal positive cells shown in bar graph in **(D)**. Cell proliferation capability was also determined by Cell Counting Kit-8 (CCK-8) method and time-course growth was plotted for both young and aged MSCs **(E)**. SIRT1 mRNA expression level was quantified by reverse-transcription quantitative-PCR for young and aged MSCs with GAPDH used as a control **(F)**. Western blot was performed with a representative protein bands shown in **(G)** and quantified in bar graph **(H)**. SIRT1 activity was also measured and expressed as fold changes shown in bar graph **(I)**. All the data are expressed as mean ± *SD* (three independent experiments, *n* = 5 rats/group). ^*^implies *P* < 0.05, compared to young MSCs.

The immunophenotype of MSCs from young and aged rats were assessed by flow cytometry using specific cell surface antigens. Both young and aged MSCs expressed the MSCs markers CD29, CD44, CD73, CD90, and CD105 and were negative for the markers CD31, CD34, and CD45 (Figure 1A). Similar plastic adherence was observed for both MSCs that were isolated from young and aged rats. Though young and aged MSCs both could differentiate toward adipogenic and osteogenic lineage, the differentiation potential of aged MSCs was impaired compared to young counterparts (Figure S1).

Senescence-associated β-galactosidase (SA-β-gal) is universally accepted as a marker for senescent cells (Kuilman et al., 2010; Sikora et al., 2011; Campisi, 2013; Sikora, 2013). To ascertain age-related cellular senescence alteration in MSCs, SA-β-gal activity was analyzed in MSCs obtained from young and aged rats at the same passage. Compared with young counterparts, the percentage of β-gal positive cells and the staining intensity increased significantly in MSCs from aged rats (Figures 1B–D). Arrested growth is the defining characteristic of a senescent cell (Campisi, 2013), therefore, we assessed the proliferation rate for both young and aged MSCs by Cell Counting Kit-8 (CCK-8) methods at the same passage. There were no significant differences in the absorbance between the two groups during the first 3 days (Figure 1E). However, the absorbance was significantly decreased in aged MSCs compared with young counterparts 3 days later (Figure 1E). These findings reveal the existence of age-related up-regulated cellular senescence in MSCs, which is also evidenced by a down-regulated cellular proliferation rate.

### Age associated alterations of SIRT1 expression and activity in MSCs

To examine whether SIRT1 expression was altered in MSCs from aged compared with young donors, we also analyzed SIRT1 expression in MSCs. Previous studies reported that SIRT1 was expressed in MSCs from young donors (Yuan et al., 2012). Our data demonstrated that not only the mRNA expression level of SIRT1 in aged MSCs was three-fold lower than young MSCs (Figure 1F), and the protein level of SIRT1 was also significantly reduced in aged MSCs compared to young counterparts (Figure 1G,H). Additionally, the enzyme activity of SIRT1 in aged MSCs was 60% lower than that of the young MSCs (Figure 1I). Thus, our data indicate that both the expression and the activity of SIRT1 are significantly decreased in MSCs from aged donors.

### SIRT1 modulates MSCs senescence and proliferation

**Figure 2:**
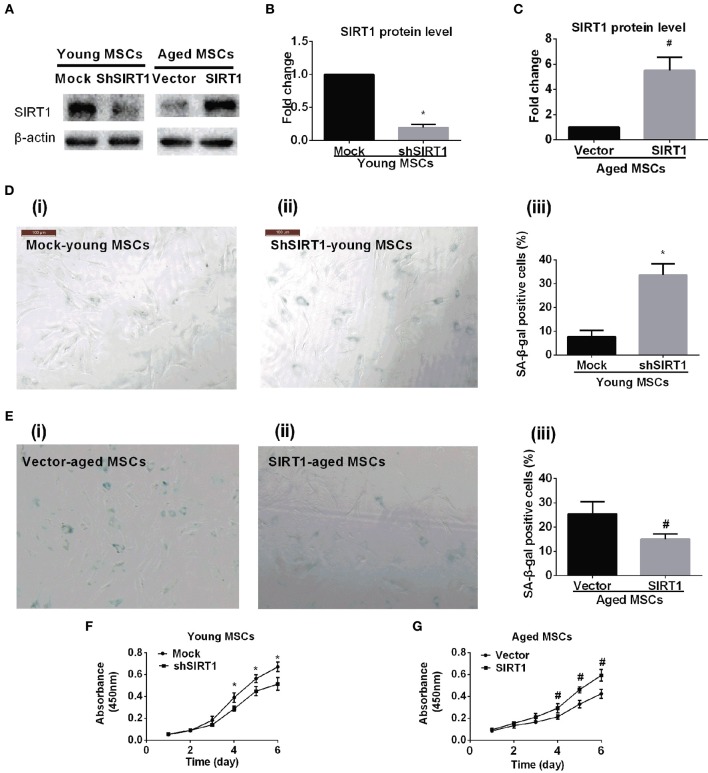
**SIRT1 mitigates age-related senescence of MSCs**. SIRT1 protein levels in genetically modified young and aged MSCs were evaluated by Western Blot. Representative protein bands were shown in **(A)** and quantified in bar graph **(B,C)**. Representative images of SA-β-gal staining for both young MSCs that were transfected with either mock- or shSIRT1-vectors (shown in **D(i)** and **D(ii)**, respectively) and aged MSC transfected with either empty vector or SIRT1 overexpressing (shown in **E(i)** and E**(ii)**, respectively), with quantification shown in bar graph **D(iii)** and **E(iii)**, respectively). Representative time-course growth of young (mock- and sh-SIRT1) **(F)** and aged (vector- and SIRT1-) **(G)** MSCs were also shown. Data are expressed as mean ± *SD* (three independent experiments, *n* = 5 rats/group). ^*^Implies *P* < 0.05, compared to mock-young MSCs. #, *P* < 0.05, compared to vector-aged MSCs.

To further elucidate whether the age-related down-regulation of SIRT1 expression and activity plays a role in MSC senescence, we tested whether gain or loss of SIRT1 expression modulated the senescence process of MSCs. We transduced young MSCs with lentiviral vectors expressing SIRT1 shRNA or nonsense shRNA. The transfection efficiency of lentivirus vector for genetically modified MSCs was evaluated by enhanced green fluorescent protein (EGFP). A representative EGFP expression is shown in Figure S2. Reduction of SIRT1 protein levels in genetically modified MSCs was evaluated by Western Blot (Figures 2A,B). Inhibition of SIRT1 expression by shRNA in young MSCs resulted in a remarkable increase in percentage of β-gal staining positive cells and its overall intensity of staining (Figure 2D). In line with this, a significantly reduction in cellular proliferation was observed when SIRT1 was silenced in young MSCs (Figure 2F).

Next, we asked whether enhanced expression of SIRT1 in aged MSCs affected cellular senescence and proliferation. Aged MSCs were transduced with lentiviruses that expresses rat SIRT1 directed by a CMV promoter. The transfection efficiency of lentivirus vector for genetically modified MSCs was evaluated by EGFP (Figure S2). Increased expression of SIRT1 protein in genetically modified MSCs was confirmed by Western Blot (Figures 2A,C). Compared with lentivirus vector control, up-regulated expression of SIRT1 in aged MSCs resulted in a decrease in the percentage of β-gal positive cells and its intensity of staining (Figure 2E). In addition, the up-regulated SIRT1 protein level was also associated with improved proliferation as shown by changes in absorbance in the CCK8 assay, suggesting that reversed SIRT1 could rescue the proliferation potential of aged MSCs (Figure 2G).

### SIRT1 mediates senescence through modulation of P16 and P21 expression in MSCs

**Figure 3:**
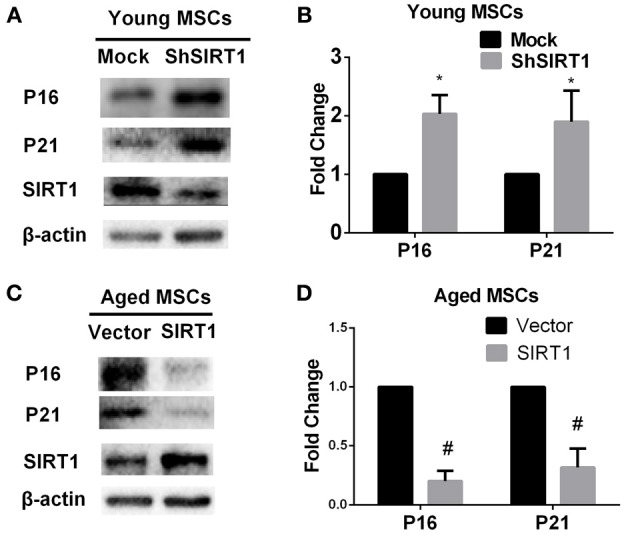
**SIRT1 positively regulates P16 and P21 expression in MSCs**. SIRT1 knockdown in young MSCs was associated with increases in P16 and P21 expression, with representative protein bands shown in **(A)** and quantified in a bar graph shown in **(B)**. In contrast, SIRT1 overexpression in aged MSCs decreases P16 and P21 expressions. Representative western blot images **(C)** and the quantitative analysis results **(D)** were shown. Data are expressed as mean ± *SD* (three independent experiments, *n* = 5 rats/group). ^*^Implies *P* < 0.05, compared to mock-young MSCs. #, *P* < 0.05, compared to vector-aged MSCs.

P16 and P21 are both implicated as mediators of cellular senescence (Kuilman et al., 2010). To explore whether P16 and P21 play roles in SIRT1-mediated actions on MSC senescence, we evaluated the expression of P16 and P21 at the protein level in young and aged MSCs transduced with SIRT1 expression vectors. We found that both P16 and P21 were significantly enhanced when SIRT1 was silenced in young MSCs (Figures 3A,B). In contrast, the expression of P16 and P21 were both significantly reduced when SIRT1 was up-regulated in aged MSCs (Figures 3C,D). These data indicate that the expression of P16 and P21 are both negatively regulated by SIRT1 in MSCs.

### SIRT1 protect telomere DNA damage in MSCs

**Figure 4:**
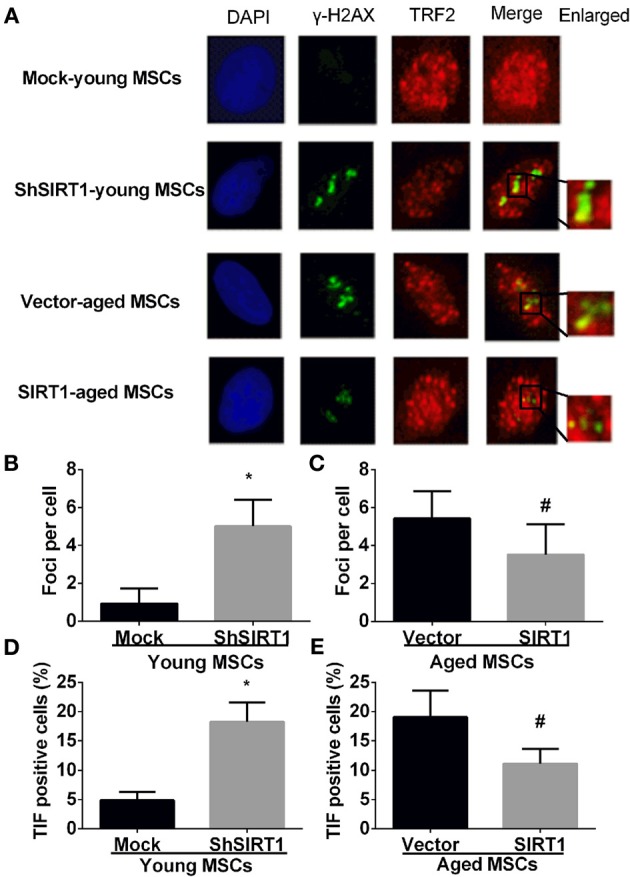
**SIRT1 protects telomere DNA damage in MSCs**. Representative immunofluorescence images of young (mock- and shSIRT1-) and aged MSCs (vector- and SIRT1) stained with γ-H2AX (Green), TRF2 (Red), and DAPI (Blue) **(A)**. Quantitative analysis of γ-H2AX foci in young (mock- and shSIRT1-) **(B)** and aged MSCs (vector- and SIRT1-) **(C)** were shown. A cell was considered TIF positive if ≥50% of its γ-H2AX foci were associated with TRF2 (as shown in merged image). Quantitative analysis of TIF positive cells in young (mock- and shSIRT1-) **(D)** and aged MSCs (vector- and SIRT1-) **(E)** were shown. Data are expressed as mean ± *SD* (three independent experiments, *n* = 5 rats/group). ^*^Implies *P* < 0.05, compared to mock-young MSCs. #, *P* < 0.05, compared to vector-aged MSCs.

P16 and P21 have important roles in driving senescence in response to DNA damage (Kuilman et al., 2010). In contrast, DNA damage increases with aging in MSCs (Asumda and Chase, 2011) and SIRT1 can positively regulate DNA repair (Jeong et al., 2007; Yuan et al., 2009; Luna et al., 2013). Thus, it is logical to postulate that increased SIRT1 prevents age-related DNA damage in MSCs. Therefore we assessed the DNA damage response when SIRT1 was manipulated in young and aged MSCs. The mean level of γ-H2AX foci, a marker of DNA damage, was significantly increased when SIRT1 was silenced in young MSCs (Figures 4A,B); in contrast, γ-H2AX foci was significantly decreased in aged MSCs when SIRT1 was over-expressed (Figures 4A,C). To further analyze whether SIRT1 action on DNA damage was related to telomere changes, we performed immunofluorescence staining of telomere induced foci (TIF) assay. A cell was considered TIF positive if ≥50% of its γ-H2AX foci merged with TRF2 (Alves et al., 2010; Brugat et al., 2010). As shown in Figures 4A,D, TIF positive cells in SIRT1-depleted young MSCs were significantly increased compared with mock-transfected young MSCs (Figures 4A,D). On the other hand, significant down-regulation of TIF positive cells was observed when SIRT1 was enhanced in aged MSCs (Figures 4A,E). This indicates that SIRT1 protects against DNA damage by modulation of the function of telomeres. Thus, our data suggest that SIRT1 decreased age-related DNA damage through modulation of telomere function in MSCs.

### SIRT1 modulates telomerase activity in MSCs

**Figure 5:**
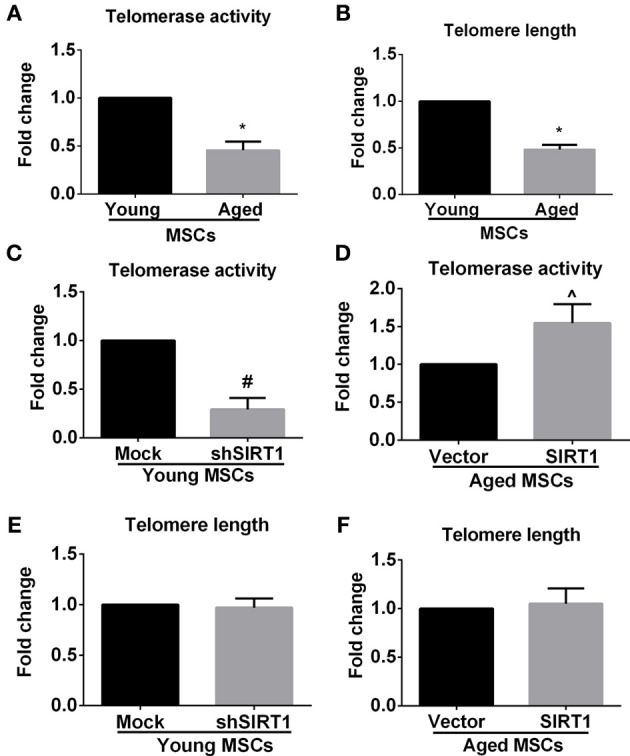
**SIRT1 modulates telomerase activity, but not telomere length in MSCs**. Quantitative analysis of age-related alterations of telomerase activity **(A)** and telomere length **(B)** in MSCs was shown. Quantitative analysis of telomerase activity in young (mock- and shSIRT1-) **(C)** and aged (vector- and SIRT1-) MSCs **(D)** was also shown. Quantitative analysis of telomere length in young (mock- and shSIRT1-) **(E)** and aged (vector- and SIRT1-) **(F)** MSCs was shown. No siginificant change in telomere length was observed when SIRT1 was manipulated in young or aged MSCs. Data are expressed as mean ± *SD* (three independent experiments, *n* = 5 rats/group). ^*^Signifies *P* < 0.05, compared to young MSCs. #, *P* < 0.05, compared to mock-young MSCs. ^, *P* < 0.05, compared to vector-aged MSCs.

Previous studies showed that telomere attrition could trigger telomere dysfunction, activate DNA damage and consequently induce cellular senescence (Palm and de Lange, 2008; Stewart et al., 2012). Telomere attrition might be closely related to impaired telomerase activity since telomere elongation is primarily catalyzed by telomerase in MSCs (Serakinci et al., 2008). Therefore we evaluated age-related alterations of telomerase activity and telomere length in MSCs. We discovered that telomerase activity and telomere length were both compromised in aged MSCs compared with young counterparts (Figures 5A,B), which was consistent with the age-associated decrease in SIRT1 expression. This raised the possibility that SIRT1 might have a role in modulating telomerase activity and telomere length. We next analyzed the telomerase activity and telomere length changes when SIRT1 was manipulated in young and aged MSCs, respectively. Telomerase activity was inhibited and induced when SIRT1 was silenced in young (Figure 5C) or overexpressed in aged MSCs (Figure 5D), respectively, which indicated that telomerase activity can be positively modulated by SIRT1. In addition, we also found that TERT expression at the mRNA level was decreased when SIRT1 was inhibited in young MSCs and was increased when SIRT1 was up-regulated in aged MSCs (Figure S3). Surprisingly, however, no significant variations in telomere length were observed when SIRT1 was either down-regulated in young MSCs (Figure 5E) or up-regulated in aged MSCs (Figure 5F). These data reveal that SIRT1 positively modulates the expression and activity of telomerase although telomere length was not significantly affected.

### SIRT1 protect telomere function through modulation of TPP1

**Figure 6:**
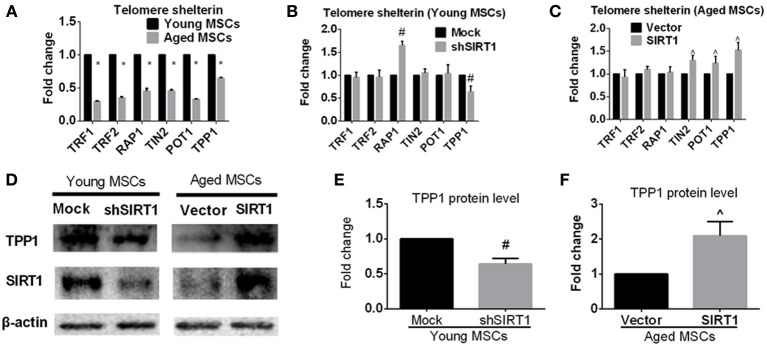
**SIRT1 positively modulates TPP1 expression in MSCs**. **(A)** Age-related changes in mRNA levels of each telomere shelterin components including TRF1, TRF2, RAP1, TIN2, POT1, and TPP1 in MSCs was quantified. The mRNA expression levels were quantified when SIRT1 was manipulated in young (mock- and shSIRT1-) **(B)** and aged (vector- and SIRT1-) MSCs **(C)**. Representative immunoblot images **(D)** and the quantitative analysis results **(E,F)** demonstrated changes in TPP1 protein level in young (mock- and shSIRT1-) and aged (vector- and SIRT1-) MSCs. Data are expressed as mean ± *SD* (three independent experiments, *n* = 5 rats/group). ^*^Implies *P* < 0.05, compared to young MSCs. #, *P* < 0.05, compared to mock-young MSCs. ^, *P* < 0.05, compared to vector-aged MSCs.

In addition to telomere attrition, telomere dysfunction can also activate the DNA damage response pathway (De Lange, 2005; Palm and de Lange, 2008; Stewart et al., 2012). Mammalian telomeres are bound by shelterin, a specialized complex composed of six core proteins, i.e., TRF1, TRF2, RAP1, TIN2, POT1, and TPP1. Shelterin plays an important role in determining the structure of the telomere terminus, generation of t-loops, and telomerase-based telomeric DNA synthesis (De Lange, 2005). It has been demonstrated that the impairment of one component of shelterin negatively influenced shelterin-induced telomere maintenance and protection (De Lange, 2005; Palm and de Lange, 2008; Stewart et al., 2012), indicating that all components of shelterin are essential for telomere function. To explore the effect of age on the expression of shelterin, we assessed the expression of shelterin components in young and aged MSCs. We found that all members of shelterin were decreased in aged MSCs compared with their young counterparts (Figure 6A). To further elucidate whether age-related alteration of SIRT1 expression was responsible for down-regulated expression of shelterin components, we analyzed the mRNA expression levels of shelterin components when SIRT1 was manipulated in young and aged MSCs. RT-PCR results showed that inhibition of SIRT1 expression by shRNA in young MSCs significantly enhanced the expression of TPP1 and RAP1 whereas the expression of TRF1, TRF2, TIN2, and POT1 were not influenced (Figure 6B). On the other hand, forced expression of SIRT1 in aged MSCs contributed to a significant up-regulation of TPP1, POT1 and TIN2 expression without affecting the expression level of TRF1, TRF2, and RAP1 (Figure 6C). Thus, TPP1 is the only shelterin component that can be regulated by both down- and up-regulation of SIRT1. Western blot showed that TPP1 protein expression was consistent with its changes in mRNA levels, demonstrating a decreased in young MSCs when SIRT1 was silenced (Figures 6D,E) and an increase in aged MSCs where SIRT1 was over-expressed (Figures 6D,F). Taken together, these results show that SIRT1 may have a role in modulating telomere shelterin and TPP1 might be a key target of SIRT1 mediated protection against age-related senescence of MSCs.

## Discussion

In this study, our data demonstrated that silencing SIRT1 in young MSCs inhibited TPP1 expression and telomerase activity and induce the DNA damage response, which is consistent with an increase of cellular senescence and decreased cellular proliferation. A reversal of these changes was observed in aged MSCs with forced SIRT1 over-expression.

Tissue-specific changes of SIRT1 expression have been reported during aging. For example, while SIRT1 expression increased in skeletal muscle, liver, heart, kidney and lung (Koltai et al., 2010; Braidy et al., 2011), it was significantly decreased in hippocampus with aging (Quintas et al., 2012). Although a previous study reported no significant difference in SIRT1 expression in MSCs obtained from 4-week-old and 15-month-old male C57BL/6 mice (Li et al., 2011), our data is in sharp contrast and clearly demonstrates significantly decreased SIRT1 expression during aging of rodent MSCs. Although we cannot account for this discrepancy, it might be related to mouse vs. rat or differences in the experimental conditions. It has been shown that SIRT1 expression decreases with increased passage number of young MSCs (Yuan et al., 2012); in results not shown we observed similar trends of SIRT1 expression in cultured aged MSCs. Therefore cell passage and culture time *in vitro* may be a critical factor when comparing different experimental groups. Thus, our data support the notion that SIRT1 expression decreases with aging.

Previous studies demonstrated that cellular proliferation capacity of MSCs is significantly compromised with aging (Stolzing et al., 2008; Zhou et al., 2008; Efimenko et al., 2011; Katsara et al., 2011; Alt et al., 2012). This change is tightly correlated with the increase in the percentage of the senescent cells in the stem cell pool (Stolzing et al., 2008; Zhou et al., 2008; Alt et al., 2012). In the current study, SA-β-gal, as a widely accepted hallmark of senescent cells (Campisi, 2013; Sikora, 2013) was induced whereas cellular proliferation ability was inhibited in aged MSCs compared to young MSCs. Moreover, down-regulated SIRT1 expression in young MSCs resulted in an inhibited cellular proliferation and an increase in percentage of β-gal positive cells. The relationship between β-gal positive cells and proliferation ability was also seen in aged MSCs where overexpressing of SIRT1 promoted cellular proliferation and down regulated β-gal positive cells. Thus, our data suggest that during aging, the β-gal expression is closely associated with proliferation capability, and importantly manipulation of SIRT1 expression can modulate these biological processes. Thus, our data not only provide evidence that SIRT1 is down-regulated during aging, but also that SIRT1 plays a key role in senescence process of aged MSCs.

The expression of P16 and P21 is increased in aged MSCs (Stolzing et al., 2008; Zhou et al., 2008; Alt et al., 2012) and *in vitro* culture (Ksiazek, 2009). A reduction in P16 (Shibata et al., 2007) or P21 (Yew et al., 2011) expression in MSCs can improve *in vitro* cellular senescence and senescence-related changes. In an *in vitro* study looking at senescent process in MSCs, Yuan and coworkers (Yuan et al., 2012) observed that SIRT1 silencing leads to increases in both P16 and P21 expression in adipose tissue-derived MSCs, however only P16, but not P21, was enhanced in bone marrow-derived MSCs, suggesting that P16 and P21 are differentially regulated during aging in MSCs isolated from different tissue sources. Interestingly, our data showed that both P16 and P21 expression are negatively correlated with SIRT1 expression in MSCs, indicating that P16 and P21 are both involved in SIRT1 mediated protection against DNA damage during MSCs senescence. Our data thus further support the previous studies demonstrating that SIRT1 can enhance DNA repair activity (Jeong et al., 2007; Li et al., 2008; Yuan et al., 2009; Dobbin et al., 2013) which could be attributed to SIRT1-induced deacetylation of histones and non-histones, including histone H3 lysine 56 (H3K56) (Yuan et al., 2009), class I histone deacetylase (HDAC1) (Dobbin et al., 2013), Ku70 (Jeong et al., 2007), and WRN (Li et al., 2008). And the changes in both P16 and P21 can reflect the effects of SIRT1 on mediating age-related DNA damage of MSCs.

SIRT1 action on telomere length has been reported previously. The single-nucleotide polymorphism of SIRT1 gene has significant influence on leukocyte telomere length (Kim et al., 2012). Moreover, overexpression of SIRT1 in mice resulted in significantly longer telomere whereas the deficiency of SIRT1 expression in mice contributed to a pronounced shorter telomere length (Palacios et al., 2010), suggesting a critical regulatory role of SIRT1 in controlling telomere length. Unexpectedly, however, no significant alteration in telomere length was observed when SIRT1 expression was manipulated in young or aged MSCs in our present study. This may be explained by the fact that the limited cell culture time after SIRT1 manipulation is not sufficient to induce obvious change in telomere length. Nevertheless, telomerase activity was closely regulated by SIRT1 expression and this is likely to play an important role in protecting against DNA damage during the aging process. Interestingly, our further studies demonstrated that the telomere length of MSCs decreased in SIRT1 knockdown, and increased in SIRT1 overexpression respectively with a longer cell culture time (passage 8 after manipulation) (Figure S4), indicating that the telomere length of MSCs *in vitro* can be regulated by SIRT1 in a longer cell culture.

TPP1, a crucial component of shelterin, plays a critical role in telomere maintenance since knockdown of TPP1 inhibits shelterin formation (O'Connor et al., 2006). Previous studies revealed that the expression of TPP1 might be modulated by SIRT1. Chen et al. (2011) have demonstrated that knockdown of SIRT1 in hepatocellular carcinoma (HCC) cells inhibited TPP1 expression and induced cellular senescence whereas up-regulated expression of TPP1 in SIRT1-depleted HCC cells improved cellular senescence, strongly suggesting that TPP1 was closely involved in SIRT1-mediated anti-senescence effects in HCC cells. In line with this study, we also found that TPP1 was positively modulated by SIRT1 in both young or aged MSCs. More importantly, in our study TPP1 was the only component of shelterin that was manipulated by either silencing or over-expressing SIRT1, and might be the main mediator of SIRT1 action on age-related senescence of MSCs. TPP1 does not contact with telomeric DNA directly, instead, it forms a complex with POT1, another shelterin component that binds the single-stranded overhang immediately at the end of chromosomes. Therefore, the beneficial effects of TPP1 on telomere maintenance are mainly mediated through POT1. On the other hand, TPP1 was indispensable for localization of POT1 to the telomeres (Liu et al., 2004; Kibe et al., 2010) and for maintaining the structure and function of the telomere. Further research indicated that the interactive actions of TPP1/POT1 on telomere maintenance also requires TIN2 (Kibe et al., 2010), which is a crucial linkage connecting TPP1/POT1 complex to TRF1 and TRF2 on the double-stranded telomeric DNA, supporting the important roles played by each of these telomere shelterin components (O'Connor et al., 2006). Here we showed that POT1 and TIN2 were both up-regulated when SIRT1 was over-expressed in aged MSCs, however, they did not show significant responses to a deletion of SIRT1 in young MSCs. Neither, did we demonstrate any changes in TRF1 and TRF2 expressions when SIRT1 expression was manipulated in MSCs. Interestingly, we found that RAP1 expression at the RNA level only exhibited a significant increase when SIRT1 was depleted in young MSCs, but showed no significant changes when SIRT1 was overexpressed in aged MSCs. It will be important to determine how these patterns of changes in shelterin components are regulated by SIRT1 activity and how this integrates with SIRT1 regulation of MSC senescence.

To date, the influence of SIRT1 on telomerase activity is not fully understood. The available information suggests that SIRT1 modulates telomerase in a cell type dependent manner. For example, inhibition of SIRT1 in human BJT diploid fibroblasts and HeLa cells leads to significant augmentation in telomerase activity (Narala et al., 2008). In contrast, in mouse embryonic fibroblasts, a proficient telomerase complex is present to maintain longer telomeres only when SIRT1 was overexpressed (Palacios et al., 2010). It has been shown that the regulation of SIRT1 on telomerase activity was mainly mediated by TERT (Narala et al., 2008). Inhibition of SIRT1 in human BJT diploid fibroblasts and HeLa cells leads to a small increase in TERT mRNA level; this modulation of TERT protein by SIRT1 was also observed in human umbilical cord fibroblasts (Yamashita et al., 2012) and hematocellular carcinomas (Chen et al., 2011). The present study showed that SIRT1 knockdown in young MSCs induced TERT expression and telomerase activity whereas SIRT1 overexpression in aged MSCs inhibited TERT expression and telomerase activity, thus further suggesting that SIRT1 regulates age-related TERT expression and telomerase activity in MSCs. In these studies we did not test whether SIRT1 modulates post-translation regulation of TERT, a regulation that has been reported previously (Narala et al., 2008).

The regulatory role of the shelterin complex on telomerase is attracting intensive interest. Results show that each shelterin component exhibits a dampening effect on telomerase activity since inhibition of even one shelterin component promotes telomerase-mediated telomere elongation (Palm and de Lange, 2008). Conflicting data also exist demonstrating that the shelterin components may have a promotive effect on telomerase for which TPP1 was identified as a critical factor. It has been shown that the effects of TPP1 on telomerase activity might depend on the interaction between TPP1 and the G100 region of TERT (Zaug et al., 2010). Considering that the expression of TPP1 could be positively modulated by SIRT1 in our experiments, it is logical to reason that the positive role of SIRT1 on telomerase activity might be mediated by TPP1. In addition, the beneficial effects of TPP1 on telomere maintenance were mainly mediated by POT1 through the TPP1-POT1 complex. This TPP1-POT1 association could not only up-regulate the activity and processivity of telomerase (Wang et al., 2007; Latrick and Cech, 2010) but also modulate the access of telomerase to telomeric DNA (Xin et al., 2007), indicating the critical role of POT1/TPP1 interaction in telomerase activity. However, this notion was challenged by a recent study that demonstrated that it is the TIN2, but not POT1, that is necessary for recruitment of telomerase to telomeres by TPP1(Abreu et al., 2010). Further studies are warranted to clarify the role of TPP1, POT1, and TIN2 in SIRT1 action on telomerase activity in MSCs during aging process.

In summary, we show that SIRT1 is biologically down-regulated in aged MSCs and this parallels cellular senescence and impaired proliferation capability. In addition, knockdown of SIRT1 in young MSCs recapitulated the cellular senescence process observed in aged MSCs, whereas overexpression of SIRT1 attenuated this biological aging process, leading to an enhanced MSC proliferation. More importantly, we further demonstrated that manipulation of SIRT1was closely associated with changes in P16 and P21 expression suggesting that SIRT1 protects against DNA damage and regulates telomere aging through TPP1. Our results confirm an important role of SIRT1 in mitigating age-related MSCs senescence and provide evidence for the notion that age-associated MSCs senescence can be modulated by SIRT1. Therefore SIRT1 might be a novel target for reversing age-induced alteration in MSCs.

## Author contributions

Conceived, designed and performed the experiments: Huiqiang Chen, Xianbao Liu, Wei Zhu, Han Chen, Xinyang Hu, Zhi Jiang, Yinchuan Xu, Lihan Wang, Yu Zhou, Panpan Chen, Na Zhang, Dexing Hu, Ling Zhang, Yaping Wang, Qiyuan Xu, Rongrong Wu, Hong Yu, and Jian'an Wang. Analyzed the data and wrote the manuscript: Huiqiang Chen, Xianbao Liu, Wei Zhu, and Jian'an Wang.

### Conflict of interest statement

The authors declare that the research was conducted in the absence of any commercial or financial relationships that could be construed as a potential conflict of interest.

## Supplementary material

Figure S1 **Adipogenic and osteogenic potential of young and aged MSCs**. Representative morphology of young and aged MSCs, the adipogenic differentiation stained with Oil red O while the osteogenic differentiation identified by alkaline phosphatase staining.

Figure S2 **Representative images of EGFP expression in young [mock- (A) and shSIRT1- (B)] and aged [vector- (C)and SIRT1- (D)] MSCs were shown**.

Figure S3 **SIRT1 positively modulates telomerase reverse transcriptase (TERT) expression in MSCs**. Quantitative analysis of TERT in young (mock- and shSIRT1-) **(A)** and aged (vector- and SIRT1-) **(B)** MSCs was shown. Data are expressed as mean ± *SD* (three independent experiments, *n* = 5 rats/group). ^*^, *P* < 0.05, compared to mock-young MSCs. #, *P* < 0.05, compared to vector-aged MSCs.

Figure S4 **SIRT1 positively regulates the telomere length of MSCs *in vitro* in a longer cell culture**. Quantitative analysis of telomere length in young (mock- and shSIRT1-) **(A)** and aged (vector- and SIRT1-) **(B)** MSCs was shown. Data are expressed as mean ± *SD* (three independent experiments, *n* = 5 rats/group). ^*^, *P* < 0.05, compared to mock-young MSCs. #, *P* < 0.05, compared to vector-aged MSCs.
