# Supplementary material for: SIRT1 ameliorates age-related senescence of mesenchymal stem cells via modulating telomere shelterin
Source: Front Aging Neurosci. 2014 Jun 3;6:103. doi: 10.3389/fnagi.2014.00103 (PMC4042159; doi:10.3389/fnagi.2014.00103)
Supplement: Figure S1 — Adipogenic and osteogenic potential of young and aged MSCs. Representative morphology of young and aged MSCs, the adipogenic differentiation stained with Oil red O while the osteogenic differentiation identified by alkaline phosphatase staining. [file DataSheet1.DOCX]

Figure S1

Figure S2

Figure S3

Figure S4
